# The Short Anxiety Screening Test in Greek: translation and validation

**DOI:** 10.1186/1744-859X-9-1

**Published:** 2010-01-05

**Authors:** Ilias A Grammatikopoulos, Gary Sinoff, Athanasios Alegakis, Dimitrios Kounalakis, Maria Antonopoulou, Christos Lionis

**Affiliations:** 1 Clinic of Social and Family Medicine, Department of Social Medicine, University of Crete, Heraklion, Greece; 2 Department of Geriatrics, Carmel Medical Center, Haifa, Israel; 3 Biostatistics Laboratory, Department of Social Medicine, Faculty of Medicine, University of Crete, Greece; 4 Health Center of Anogia, Anogia, Crete, Greece; 5 Health Center of Spili, Spili, Crete, Greece

## Abstract

**Background:**

The aim of the current study was to assess the reliability and validity of the Greek translation of the Short Anxiety Screening Test (SAST), for use in primary care settings. The scale consists of 10 items and is a brief clinician rating scale for the detection of anxiety disorder in older people, particularly, in the presence of depression.

**Methods:**

The study was performed in two rural primary care settings in Crete. The sample consisted of 99 older (76 ± 6.3 years old) people, who fulfilled the participating criteria. The translation and cultural adaptation of the questionnaire was performed according to international standards. Internal consistency using the Cronbach α coefficient and test-retest reliability using the intraclass correlation coefficient (ICC) was used to assess the reliability of the tool. An exploratory factor analysis using Varimax with Kaiser normalisation (rotation method) was used to examine the structure of the instrument, and for the correlation of the items interitem correlation matrix was applied and assessed with Cronbach α.

**Results:**

Translation and backtranslation did not reveal any specific problems. The psychometric properties of the Greek version of the SAST scale in primary care were good. Internal consistency of the instrument was good, the Cronbach α was found to be 0.763 (*P *< 0.001) and ICC (95% CI) for reproducibility was found to be 0.763 (0.686 to 0.827). Factor analysis revealed three factors with eigenvalues >1.0 accounting for 60% of variance, while the Cronbach α was >0.7 for every item.

**Conclusions:**

The Greek translation of the SAST questionnaire is comparable with that of the original version in terms of reliability, and can be used in primary healthcare research. Its use in clinical practice should be primarily as a screening tool only at this stage, with a follow-up consisting of a detailed interview with the patient, in order to confirm the diagnosis.

## Background

Anxiety remains one of the most common mental problems that older individuals experience [1,2], although anxiety disorders in older people appear to remain underdiagnosed and undertreated by primary care practitioners [3,4]. The development of accurate diagnostic instruments for use in primary healthcare (PHC) remains a challenge, especially in settings with limited resources and research capacity such as Greece [5-7]. The necessity of the development for this kind of instruments for primary care settings arises from a recent review which declares that the longstanding dominance of medical perspectives in Greek health policy has been paving the way towards vertical integration, pushing aside any discussions about horizontal or comprehensive integration of care [8]. Furthermore, the use of recognised tools constitutes a necessity for the international community, not only for epidemiologic comparisons but also for quality of life improvement [9-13].

Several instruments have been translated into Greek for the identification of depression [14-16] and for anxiety disorders with self-rated instruments [17,18]. Anxiety disorders among older people seem to constitute a somewhat neglected subject in Greeceand the area needs more attention [19,20], especially because doctors have difficulties in diagnosing and managing anxiety disorders [21-23]. The Short Anxiety Screening Test (SAST) was developed to provide clinicians with a simple tool for detecting anxiety disorders in older people. It was developed and standardised in 1999 by Sinoff *et al *[24] and was considered appropriate for our study purpose for the following reasons: it is short and easy to apply in clinical settings and it is based on an interviewer-assisted self-rating scale, rendering it practical for use in everyday practice. According to the developers, the instrument can accurately and reliably identify symptoms of anxiety in older people even, and especially, in the presence of depression [24].

This article reports on the translation and validation of the SAST questionnaire and discusses several possibilities for implementation in the Greek primary healthcare setting.

## Methods

### Questionnaire

The SAST fulfils the criteria defined by the Diagnostic and Statistical Manual of Mental Disorders, fourth edition (DSM-IV) and contains questions relating to somatic symptoms, often the manifestation of anxiety in older people [25]. It includes, among others, modifications of selected, commonly recurring questions as found in other instruments. The scale consists of 10 items rated on a 4-point response scale ranging from 1 to 4 and generating scores between 10 and 40, with a higher score equalling a higher degree of anxiety. Responses include 'rarely or never', 'sometimes', 'often' and 'always' (see Additional file 1). SAST requires 10 to 15 min to administer and a total score is calculated by the sum of the grades of all questions. A score of ≥ 24 is the cut-off point for the diagnosis of anxiety, while a score of 22 to 23 reflects borderline test results.

### Study population

In all, 99 consecutive patients attending 2 rural PHC centres in Crete over a period of 2 months were recruited. The study took place during the morning shifts of two doctors. All participants agreed to complete the questionnaire. Eligibility criteria included that participants should be over 65 years old, should have given their written consent, and were free of any cognitive impairment according to the doctor's records.

At 2 weeks later, the final 26 participating persons from 1 PHC centre were selected to answer the questionnaire for a second time, and all of them agreed to do so (retest response rate 100%). This period of time is considered neither too long for a person's mental status to have changed, nor too short from the first application. The size of the retest sample (n = 26) was sufficient as suggested by Walter *et al *[26].

### Translation

Based on procedures set by the Clinic of Social and Family Medicine at the University of Crete, written permission was obtained by the original developers and also the copyright holder, to proceed with the translation and use of the tool for research purposes only. The translation and cultural adaptation of SAST was performed according to 'The Minimal Translation Criteria' [27]. Two independent bilingual physicians with advanced levels of English language and mother tongue of the Greek language translated the questionnaire into Greek (forward translation). With the contribution of a third reviewer, a reconciliation meeting was conducted to develop a consensus version (reconciliation Greek version). A psychologist, who was a native English speaker and who was blinded to the original version, retranslated the reconciliated Greek version into the source language (backtranslation). The backtranslation was sent to the developer of the original questionnaire for comparison and his suggestions were incorporated, thus formulating the revised Greek version of the SAST.

**Figure 1:**
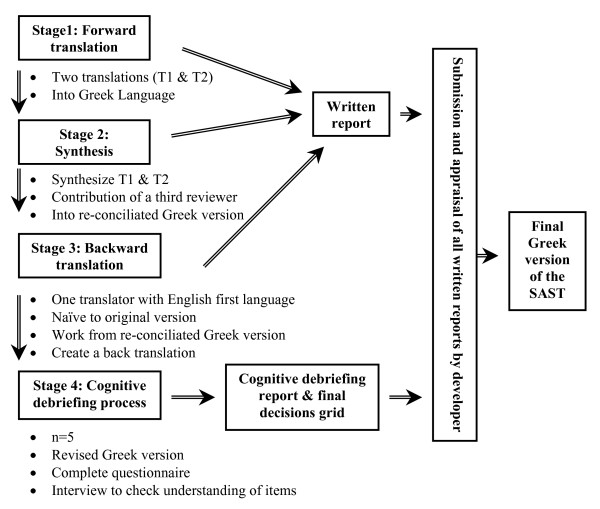
**Graphic representation of the stages of the translation process**.

A cognitive debriefing process was used for the cultural adaptation of the questionnaire as the last step of the translation procedure [27]. This process was carried out in order to identify any areas presenting linguistic problems and to assess the patient's level of understanding with the purpose of revealing inappropriate items and translation alternatives. As part of this process, the questionnaire was administered to five attendants of a PHC centre, and comments made by them were discussed in a debriefing summary and a final debriefing decision grid was sent to the developer for comments; this led to the final Greek version of the SAST. Figure 1 demonstrates the flow of the translation process.

### Statistical analysis

Descriptive characteristics (including means, SDs, frequencies and percentages) were calculated for the sociodemographic variables. For categorical data we used Pearson r, and for dichotomous discrete data the χ^2 ^statistic. For categorical data with more than two terms we used one-way analysis of variance (ANOVA) and in cases of statistical significance, a *post hoc *(Student-Newman-Keuls) analysis was performed.

### Reliability

Internal consistency and reproducibility were measured as part of the reliability testing of the translated tool [28]. Internal consistency was determined by the use of Cronbach *a*, requiring a minimum value of 0.70 for group and 0.90 for individual comparisons [29,30]. Reproducibility (test- retest reliability) is a measure of strength of association for determining stability of the questionnaire's results over time because it corrects for lack of independence between measurement intervals [28]. Reproducibility was measured by calculating the intraclass correlation coefficient (ICC) [31]. The test-retest reliability coefficient, sometimes called the stability coefficient, tests the assumption that when a characteristic is measured twice, both measures must lead to comparable results. However, test-retest reliability is only a valid indicator of the reliability of an instrument if the characteristic under study has not changed in the interval between testing and retesting. This means either a relatively stable characteristic (such as intelligence, personality, socioeconomic status) or a short time interval. A short time interval between test administrations, however, may produce biased (inflated) reliability coefficients, due to the effect of memory [32].

### Validation

A factor analysis was performed in order to determine the structure of the questionnaire and to highlight how the individual items grouped together [33,34]. The factor structure was studied by principal component analysis using Varimax with Kaiser normalisation as rotation method. A factor was considered important if its eigenvalues exceeded 1.0 [35].

### Ethics

The scientific committee of the University Hospital of Heraklion, Crete approved this study (protocol no. 12521/25/10/2006). All participants involved in the cultural adaptation and reproducibility (test-retest reliability) procedure were informed about the scope and the purpose of the study, and provided written consent.

## Results

### Study population

The study involved 99 participating individuals, with a mean age of 76 years (SD ± 6.36 years), consisting of 56 women (56.6%) and 43 men (43.4%). The age distribution was equable, since 46 persons (46.5%) were within the age range of 65 to 74 years and 53 persons (53.5%) were >75 years old (Table 1). There was no statistically significant difference when we compared the health centres and sex (χ^2 ^= 0.152, degrees of freedom (df) = 1, *P *= 0.697) or the health centres and the age distribution (χ^2 ^= 0.567, df = 1, *P *= 0.451) (Table 2).

**Table 1:** Demographic characteristics of the sample

	Number, N	Frequency, %	Mean (± SD)
Sex:			
Male	43	43.4%	76.5 (± 6.3)
Female	56	56.6%	75.6 (± 6.4)
Age distribution:			
65 to 74	46	46.5%	
≥ 75	53	53.5%	
Health centre:			
Spili	62	62.6%	
Anogia	37	37.4%	

**Table 2:** Comparison of the parameters of the study sample

	Health centre			
			
	Anogia, N (%)	Spili, N (%)	Total, N (%)	Pearson χ^2^
Sex:				
Female	20 (54.1%)	36 (58.1%)	56 (56.6%)	χ^2 ^= 0.152, df = 1, *P *= 0.697
Male	17 (45.9%)	26 (41.9%)	43 (43.4%)	
Age distribution:				
65 to 74	19 (41.3%)	27 (58.7%)	46 (46.5%)	χ^2 ^= 0.567, df = 1, *P *= 0.451
≥ 75	18 (34.0%)	35 (66.0%)	53 (53.5%)	
Total N (%)	37 (100.0%)	62 (100.0%)	99 (100.0%)	χ^2 ^= -0.382, df = 97, *P *= 0.704

When the total scores for SAST were examined, the test results proved negative for 58.6% (N = 58), borderline for 12.1% (N = 12), and positive for 29.3% (N = 29) (Table 3).

**Table 3:** Comparison of the Short Anxiety Screening Test (SAST) results (analysis of variance (ANOVA))

Results	N (%)	Mean (± SD)	Minimum	Maximum	ANOVA		
Negative test	58 (58.6%)	17.6 (± 2.3)	12	21	F = 188,281, df = 2, *P *< 0.0001		
Borderline test	12 (12.1%)	22.3 (± 0.5)	22	23			
Positive test	29 (29.3%)	28.5 (± 3.3)	24	36			
Total	99 (100%)	21.3 (± 5.5)	12	36			

The mean score for older people with negative results was 17.6 (SD ± 2.28), whilst for those with a positive result the mean score was 28.5 (SD ± 3.24). The application of ANOVA identified a statistically significant difference between the scores (*P *< 0.0001, F = 188,281) (Table 3). *Post hoc *analysis showed that the SAST score differed at the significance level *P *< 0.0001.

The total mean score of the SAST for the study population as a whole was 21.3 (SD ± 5.5; min 12, max 36). The mean score for women was 22.8 (SD ± 5.8) and for men 19.5 (SD ± 4.3). With the use of t test for independent samples, this difference was found to be statistically significant (t = 3.105, df = 97, *P *= 0.002). In contrast, there was no statistically significant difference when we compared the mean scores across age distribution (t = 0.837, df = 97, *P *= 0.404) or for the individual health centres (t = -0.382, df = 97, *P *= 0.704) (Table 4).

**Table 4:** Comparison of the Short Anxiety Screening Test (SAST) results for sex, age distribution and health centres

	Frequency, N	SAST score, mean (± SD)	t Test
Sex:			
Male	56	22.8 (± 5.8)	t = 3.105, df = 97, *P *= 0.002
Female	43	19.5 (± 4.3)	
Age distribution:			
65 to 74	46	21.8 (± 5.5)	t = 0.837, df = 97, *P *= 0.404
≥ 75	53	20.9 (± 5.5)	
Health centre:			
Spili	37	21.1 (± 6.1)	t = 0.382, df = 97, *P *= 0.704
Anogia	62	21.5 (± 5.1)	

### Translation

The translation procedures did not reveal any specific problems. The developers of the SAST made some comments on three of the backtranslated questions where minor issues were identified. These concerned the interpretation of the word 'irritable' (question 8), the differentiation of the expression 'back pain' (question 6) and the interpretation of the word 'palpitations' (question 7), emphasising the somatic complaints of older people. These comments were taken into account when finalising the Greek reconciliated version of the SAST.

During cultural adaptation, the questionnaire was found to be overall comprehensible and easy to understand, according to comments from older people. The only linguistic problem concerned question 8, where all respondents proposed to change the Greek word for 'irritable' into a less obscure word that would be more easily understood by the respondents. Their recommendation was discussed and incorporated into the final Greek translation of the questionnaire.

Feedback from the doctors demonstrated that the questionnaire was comprehensible, easy and quick (approximately 10 min) to use, and that it could be used in everyday clinical practice for primary assessment, while interviewing the patients regarding mental health issues.

### Reliability and validity

The SAST questionnaire showed a very good overall internal consistency (α value: 0.763, 95% CI 0.71 to 0.82, *P *< 0.001) for individual comparison. The overall Cohen κ coefficient for reproducibility (test-retest reliability) was 'very good' (0.930, 95% CI 0.918 to 0.942, *P *< 0.0001) and ICC (95% CI) for reproducibility was found to be 0.763 (95% CI 0.686 to 0.827) [25]. The Wilcoxon signed ranks test showed that there was no statistically significant difference between the total of questions (z = 0.676, *P *= 0.499), as in the comparison for each question separately between the two applications of questionnaire (N = 26), with values oscillated from z = 0.0 (*P *= 1.0) in question 3, to z = 1.134 (*P *= 0.257) in question 9. The results are illustrated in Table 5.

**Table 5:** Short Anxiety Screening Test (SAST) reproducibility (test-retest reliability)

Question	First application (test), (N = 26), mean ± SD	Second application (retest), (N = 26), mean ± SD	z^a^, *P *value
Question 1	2.12 ± 0.816	2.15 ± 0.784	z = 1.000, *P *= 0.317
Question 2	1.92 ± 0.977	1.96 ± 0.958	z = 1.000, *P *= 0.317
Question 3	2.15 ± 0.543	2.15 ± 0.543	z = 0.000, *P *= 1.00
Question 4	2.42 ± 0.758	2.38 ± 0.697	z = 0.577, *P *= 0.564
Question 5	2.58 ± 0.857	2.62 ± 0.752	z = 0.577, *P *= 0.564
Question 6	1.54 ± 0.859	1.58 ± 0.857	z = 1.000, *P *= 0.317
Question 7	1.27 ± 0.533	1.31 ± 0.549	z = 1.000, *P *= 0.317
Question 8	1.69 ± 0.838	1.62 ± 0.697	z = 1.000, *P *= 0.317
Question 9	2.19 ± 1.167	2.31 ± 1.087	z = 1.134, *P *= 0.257
Question 10	1.65 ± 0.797	1.58 ± 0.758	z = 0.632, *P *= 0.527
Total	19.58 ± 3.489	19.69 ± 3.541	z = 0.676, *P *= 0.499

^a^Wilcoxon signed rank test.

Exploratory factor analysis indicated three factors with eigenvalues over 1.0. Those factors were responsible for 60% of variance and rotation converged in three iterations (Table 6). At the same time, for the control of crosscorrelation of items among them using the interitem correlation matrix method, analysis showed that all questions correlated very well, as Cronbach α values were all greater than 0.7 (Table 7).

**Table 6:** Factor analysis for the symptoms: rotated component matrix for three factors

		Rotation sums of squared loadings			
		
Components		Variance of factor	Eigenvalues	Degree of explanation, %	Cronbach α
Factor I (somatic symptoms and autonomic arousal)	Item 6	0.676	2.307	23.074	0.699
	Item 7	0.761			
	Item 9	0.611			
	Item 10	0.745			

Factor II (symptoms of tension and distress)	Item 1	0.809	1.837	18.374	0.642
	Item 2	0.461			
	Item 8	0.838			

Factor III (mental state symptoms: fears and concerns)	Item 3	0.430	1.818	18.183	0.618
	Item 4	0.860			
	Item 5	0.810			

**Table 7:** Short Anxiety Screening Test (SAST) interitem correlation matrix

	Question 1	Question 2	Question 3	Question 4	Question 5	Question 6	Question 7	Question 8	Question 9	Question 10	Cronbach α if item deleted
Question 1	1.000	0.291	0.247	0.025	0.132	0.280	0.206	0.485	0.109	0.124	**0.752**
Question 2	0.291	1.000	0.437	0.117	0.350	0.242	0.318	0.291	0.247	0.290	**0.732**
Question 3	0.247	0.437	1.000	0.319	0.280	0.246	0.401	0.274	0.199	0.338	**0.729**
Question 4	0.025	0.117	0.319	1.000	0.511	0.053	0.075	0.017	0.163	-0.011	**0.770**
Question 5	0.132	0.350	0.280	0.511	1.000	0.138	0.196	0.145	0.416	0.244	**0.736**
Question 6	0.280	0.242	0.246	0.053	0.138	1.000	0.456	0.295	0.374	0.344	**0.737**
Question 7	0.206	0.318	0.401	0.075	0.196	0.456	1.000	0.078	0.294	0.414	**0.736**
Question 8	0.485	0.291	0.274	0.017	0.145	0.295	0.078	1.000	0.091	0.158	**0.753**
Question 9	0.109	0.247	0.199	0.163	0.416	0.374	0.294	0.091	1.000	0.338	**0.741**
Question 10	0.124	0.290	0.338	-0.011	0.244	0.344	0.414	0.158	0.338	1.000	**0.742**

The independent samples t test identified the SAST's ability to discriminate between older men and women, with women scoring significantly higher. Higher levels of anxiety in women have been reported in previous studies [1,2,36].

## Discussion

### Main findings

The current study suggests that the Greek version of the SAST is suitable for use in the Greek primary healthcare setting, demonstrating good internal consistency and high test-retest reliability. The factor structure of the Greek translation is similar to that reported in the literature [37]. The statistically significant difference between the total scores for older people with positive results, and for those with negative results (28,5 vs 17,6), offers further support for the validity of the questionnaire. Furthermore, the Greek version of SAST was able to discriminate between male and female patients. This result sues for the original study for the development of the SAST (25.3 vs 20.1) [24].

### Implications for practice

Accurate screening for anxiety symptoms in older populations is a crucial first step in identifying patients in need of further diagnostic procedures and treatment [38]. Although the use of self-report scales is frequent in psychiatric research, saving time for the clinician, it is also well known that these types of scales depend heavily on the cooperation and reading ability of the patient [16-18]. Our criteria was partially based on this fact when we selected the SAST, because it is an interviewer-assisted observational instrument, developed specifically for the detection of anxiety in older people, even and especially in the presence of depression, according to the developers of the original SAST questionnaire [24]. In addition, its brevity as a screening instrument (10 questions), renders it useful in everyday clinical practice and especially by primary care physicians.

Although a substantial amount of literature has addressed the overlap between depression and medical conditions [39], the same attention has not been given to anxiety disorders. Clinical ratings of anxiety severity also appear useful for older adults, although differentiation of anxiety and depression continues to be an issue of concern with regard to interpretation of scores [40].

Anxiety is one of the most common psychiatric diagnoses in primary care populations [41]. Thus, screening questionnaires are actually evaluated for their ability to detect unrecognised anxiety symptoms and disease. They are also useful for the follow-up assessment though not for an accurate diagnosis. These instruments are of particular value in primary care settings because it is clear that primary care providers fail to diagnose and treat as many as 35% to 50% of patients with anxiety disorders [42-44].

The findings from our study imply that the Greek translation of the SAST is a useful and reliable instrument for primarily detecting anxiety disorders in older patients attending Greek primary healthcare settings. The instrument is quick and easy for clinicians to use, and is easily understood by the attending patients.

### Limitations and concerns

The current study is not without certain limitations. Firstly, the study presents preliminary data and in addition the study sample was small and test-retest data was only available for 26 subjects. Full-scale validation requires the application of the scale in larger samples, and with the application of more sophisticated methodology, such as the use of borderline cases and comparison with psychiatric interview. Further testing of the SAST on a sample of psychogeriatric patients, as well as patients in long-term care facilities, those with dementia of mild severity, and also older people with general medical conditions commonly associated with anxiety symptoms, is required before the instrument can be more generally recommended for clinical practice.

We conducted a factor analysis to explore the structure of the Greek translation of the SAST, which was not applied in the original study of the SAST developers. This enabled us to identify the separate factors contributing to the composition of the questionnaire. The use of standardised instruments is important for the development of research capacity in PHC. As such, various studies have explored the use of questionnaires for measuring the frequency of health problems in primary care, and the impact of various physical conditions on the quality of life of Greek patients [45,46]. It is anticipated that the translated and validated version of SAST could be used as a practical instrument for use by primary care physicians for the identification of symptoms of anxiety, in addition to its use as a research tool.

However, we recommend that the application of the current Greek translation of SAST is restricted to its use as a screening tool, within primary care settings. Thus the SAST could be used to obtain preliminary information with regard to anxiety symptoms, which would then need to be followed-up by a detailed interview with the patient, for a diagnosis to be confirmed. This Greek version of SAST could facilitate clinical observational research in primary care and general practice, contributing to the formulation of diagnostic nomograms and particularly to the pretest probability. Furthermore, it is proposed that the Greek SAST could be used in routine care simultaneously with the Greek version of the World Health Organization WHO-5 wellbeing index. The WHO-5 is a five-item measure of wellbeing, widely used as a depression screener, with an established clinical cut-off point. The use of the two of these instruments together over time may provide useful information with regard to patients scoring below the WHO-5 cut-off point, and demonstrating anxiety as identified by SAST.

## Conclusions

The Greek translated SAST questionnaire appears to be a reliable and valid tool for screening for anxiety symptoms in older people. Due to its brevity and ease of administration, the SAST could be a useful instrument for routine practical use within Greek primary care settings.

## Competing interests

The authors declare that they have no competing interests.

## Authors' contributions

CL conceived the study design, participated in the translation of the questionnaire, formed the layout of the manuscript and co-wrote the final draft of the manuscript. GS participated with continuous consultation and co-wrote the final draft of the manuscript. IAG participated in the translation of the questionnaire, contributed in the data collection and data entry, carried out the analysis, formed the layout of the manuscript and wrote the final manuscript. AA carried out the statistical analysis and provided consultation during the validation process. DK participated in the data collection and interpretation. MA contributed in the data collection and interpretation. All authors read and approved the final manuscript.

## Supplementary Material

Additional file 1 **Short Anxiety Screening Test**. The Greek version of the questionnaire. Click here for file
