# Supplementary material for: The Short Anxiety Screening Test in Greek: translation and validation
Source: Ann Gen Psychiatry. 2010 Jan 5;9:1. doi: 10.1186/1744-859X-9-1 (PMC2819236; doi:10.1186/1744-859X-9-1)
Supplement: Additional file 1 — Short Anxiety Screening Test. The Greek version of the questionnaire. [file 1744-859X-9-1-S1.DOC]

# Additional files

**1. SAST - The Greek version of the questionnaire.**

| **ΣΥΝΤΟΜΟ ΕΡΩΤΗΜΑΤΟΛΟΓΙΟ ΑΝΙΧΝΕΥΣΗΣ ΑΓΧΟΥΣ** | | | | | | |
| --- | --- | --- | --- | --- | --- | --- |
|  | **ΕΡΩΤΗΣΕΙΣ** | **ΣΠΑΝΙΑ Ή ΠΟΤΕ** | **ΜΕΡΙΚΕΣ ΦΟΡΕΣ** | **ΣΥΧΝΑ** | **ΠΑΝΤΑ** | **ΒΑΘΜΟΙ** |
| **1** | **Αισθάνεστε εκνευρισμένος, ανυπόμονος;** | 1 | 2 | 3 | 4 |  |
| **2** | **Αισθάνεστε ότι κάτι τρομερό πρόκειται να συμβεί;** | 1 | 2 | 3 | 4 |  |
| **3** | **Ανησυχείτε για την τωρινή σας κατάσταση;** | 1 | 2 | 3 | 4 |  |
| **4** | **Αισθάνεστε ότι έχετε τον έλεγχο της ζωής σας;** | 4 | 3 | 2 | 1 |  |
| **5** | **Μπορείτε να χαλαρώσετε;** | 4 | 3 | 2 | 1 |  |
| **6** | **Υποφέρετε από πόνους στη πλάτη, πόνους στον αυχένα και από πονοκεφάλους;** | 1 | 2 | 3 | 4 |  |
| **7** | **Ιδρώνετε πολύ ή υποφέρετε από ταχυπαλμίες;** | 1 | 2 | 3 | 4 |  |
| **8** | **Είσαστε οξύθυμος;** | 1 | 2 | 3 | 4 |  |
| **9** | **Κοιμάστε καλά;** | 4 | 3 | 2 | 1 |  |
| **10** | **Υποφέρετε από ζαλάδα ή αδυναμία;** | 1 | 2 | 3 | 4 |  |
| Κλειδί: βαθμολογία≥24= θετικό αποτέλεσμα δοκιμασίας  βαθμολογία 22-23 = οριακά αποτελέσματα δοκιμασίας | | | | | | |
